# Hyperactivity of Mek in TNS1 knockouts leads to potential treatments for cystic kidney diseases

**DOI:** 10.1038/s41419-019-2119-7

**Published:** 2019-11-18

**Authors:** Zong-Ye Wu, Chun-Lung Chiu, Ethan Lo, Yuh-Ru Julie Lee, Soichiro Yamada, Su Hao Lo

**Affiliations:** 1 Department of Biochemistry and Molecular Medicine, University of California-Davis, Sacramento, CA 95817 USA; 2 Department of Plant Biology, University of California-Davis, Davis, CA 95616 USA; 3 Department of Biomedical Engineering, University of California-Davis, Davis, CA 95616 USA

**Keywords:** Cell biology, Kidney diseases

## Abstract

Cystic kidney disease is the progressive development of multiple fluid-filled cysts that may severely compromise kidney functions and lead to renal failure. TNS1 (tensin-1) knockout mice develop cystic kidneys and die from renal failure. Here, we have established TNS1-knockout MDCK cells and applied 3D culture system to investigate the mechanism leading to cyst formation. Unlike wild-type MDCK cells, which form cysts with a single lumen, TNS1-knockout cysts contain multiple lumens and upregulated Mek/Erk activities. The multiple lumen phenotype and Mek/Erk hyperactivities are rescued by re-expression of wild-type TNS1 but not the TNS1 mutant lacking a fragment essential for its cell–cell junction localization. Furthermore, Mek inhibitor treatments restore the multiple lumens back to single lumen cysts. Mek/Erk hyperactivities are also detected in TNS1-knockout mouse kidneys. Treatment with the Mek inhibitor trametinib significantly reduces the levels of interstitial infiltrates, fibrosis and dilated tubules in TNS1-knockout kidneys. These studies establish a critical role of subcellular localization of TNS1 in suppressing Mek/Erk signaling and maintaining lumenogenesis, and provide potential therapeutic strategies by targeting the Mek/Erk pathway for cystic kidney diseases.

## Introduction

Cystic kidney disease is a group of renal disorders that cause abnormal fluid-filled cysts. These diseases may be acquired from chronic renal defects or inherited due to gene mutations. The most frequent form of cystic kidney disease is the inherited polycystic kidney disease (PKD), which is a common cause of end stage renal disease (ESRD)^1^. Two common types of PKD are autosomal dominant polycystic kidney disease (ADPKD) and autosomal recessive polycystic kidney disease (ARPKD). ADPKD often results from mutations in *PKD1* or *PKD2* genes and patients usually develop signs and symptoms between the ages of 30 and 40. ARPKD is a rarer disease caused by mutations in the *PKHD1* gene and often leads to fetal or neonatal death^1,2^. Despite differences in the age of onset, disease severity, and cyst distribution of various PKDs, cyst formation commonly results from dysregulated cell proliferation and/or apoptosis, increased secretion into tubular lumen, abnormal cell–cell or cell–matrix interactions, loss of cellular polarity, and cilium dysfunction^3^. The critical roles of these events are supported by numerous studies, including phenotypic characterizations of genetically engineered mouse models^4^ and cyst formation studies using Madine-Darby Canine Kidney (MDCK) cells in 3-dimensional (3D) culture systems^5–7^. However, no effective treatment to prevent or slow down PKD progression in patients is currently available.

The human tensin family consists of four members (tensin-1, tensin-2, tensin-3, and cten) that all reside at focal adhesions^8,9^. Tensin-1 (TNS1) is also localized to cell–cell junctions^10^. All tensins contain two domains at their C-termini: the Src homology 2 (SH2) and phosphotyrosine binding (PTB) domains. Their PTB domains bind to β-integrin NPXY motifs and this direct interaction is required for maintaining β1-integrin activity^11^, which is essential for many cellular events, including cell adhesion, migration, and proliferation. The SH2 domains bind to phosphotyrosine-containing proteins, such as EGFR, c-Met, Axl, Src, Fak, p130cas, and paxillin^8,12–15^ and transduce signaling cascades mediated by protein tyrosine kinases. Tensins also regulate small GTPase signaling pathways by binding to the Rho GTPase-activating protein DLC1 (deleted in liver cancer 1)^16–18^ or Dock5, a guanine nucleotide exchange factor for the GTPase Rac^19,20^. Additionally, TNS1 interacts with actin filaments and modulates the actin cytoskeleton network^21^. These interactions provide molecular linkages between integrin receptors and the actin cytoskeleton and also mediate multiple signaling transduction pathways. These pathways modulate a host of biological events including cell adhesion, migration, proliferation, apoptosis, and differentiation^8,9,19,22^.

The role of TNS1 in the kidney has been illustrated in TNS1 knockout (KO) mouse studies. TNS1-KO kidneys are clinically and histologically normal for the first 2–3 months, then they start developing interstitial fibrosis, infiltrates, tubular dilations, and higher BUNs^23^. The renal conditions grow progressively worse, and mice die at around 10–18 months old. Cysts are only found in the kidneys but not in other tissues. However, TNS1-KO mice also develop enlarged posterior mitral leaflets with abnormal collagen and proteoglycan deposits^24^. These are characteristic features of nonsyndromic mitral valve prolapse (MVP), a common degenerative cardiac valvopathy. This finding validates the genome-wide association studies that have identified TNS1 as a risk locus for MVP^24^. Coincidentally, MVP and mitral regurgitation are very common in PKD patients^25,26^. Despite all these findings, the molecular mechanism leading to the formation of cystic kidneys and eventual renal failure caused by TNS1 deficiency remains unclear.

In this study, we have generated an in vitro TNS1-KO MDCK cell system to determine the molecular mechanism leading to cyst formation and validated the in vitro results in KO mice. With the obtained findings, we further investigated the potential therapeutic strategy for cystic kidney diseases using TNS1-KO mice.

## Materials and methods

### Reagents

Rabbit anti-TNS1 antibody was generated against human TNS1 aa1328–1339 peptide^16^. Antibodies against E-cadherin (#9121), pMEK1/2(S221) (#2338), pMEK1/2(S217/221) (#9154), Mek1/2 (#9122), pERK1/2(T202Y204) (#9101), ERK1/2 (#9194), pAkt(S473) (#9271), Akt (#9272), pGSK3β(S9) (#5558), and GSK3β (#12456) were from Cell Signaling Technology. Anti-GAPDH (#CB1001) was from Millipore. Anti-Vinculin (#693291) was from MP Biomedical. Anti-mouse or rabbit IgG HRP conjugated secondary antibodies (#7076, #7074) were from Cell Signaling Technology. Alexa Fluor secondary antibodies (488/594) and Alexa Fluor 488/594 Phalloidin were from Thermo Scientific and anti-GP135/Podocalyxin (#3F2/D8) was from the Developmental Studies Hybridoma Bank at the University of Iowa. VECTASHIELD antifade DAPI mounting solution was from Vector Labs. Mek inhibitors CI-1040 (#S1020), selumetinib (#S1008), trametinib (#S2673) were from Selleckchem, and PD98059 (#9900), U0126 (#9903) were from Cell Signaling Technology.

### Plasmids, DNA transfection, and cell culture

All constructs were generated using Gibson assembly cloning and were confirmed by DNA sequencing. The plasmid DNA was transfected into cells with Lipofectamine 2000 (Invitrogen) according to the manufacturer’s protocol. For subcellular localization studies, cells were harvested 24 h post-transfection for analysis. For establishing TNS1-KO cells, MDCK cells from ATCC were co-transfected with the Cas9 vector carrying TNS1 guide sequence (5′-ACCTGGTGTACGTCACGGAG-3′) and the donor vector containing puromycin selection cassette flanked by 5′ and 3′TNS1 homologous regions. After transfection, cells were passaged 7 times at a dilution of 1:10 (about 23 days post transfection), then cells were treated with 5 µg/ml puromycin. Stable clones were isolated and expended for further validation and analysis. Stable TNS1-KO MDCK cells were maintained in complete medium (Dulbecco’s Modified Eagle Medium containing glutamine, 4 g/L glucose, 10% fetal bovine serum, and 1% penicillin–streptomycin) supplemented with 2 µg/ml puromycin. To re-introduce wild-type TNS1 or TNS1 mutants into TNS1-KO MDCK cells, GFP-TNS1, tdTomato-TNS1, tdTomato-TNS1Δ882–1032 fusion plasmids were transfected into KO cells. Cells were selected against 750 µg/ml G418 sulfate. Stable clones were maintained in complete medium containing 250 µg/ml G418 sulfate and 2 µg/ml puromycin.

### 3D Culture and analysis

For 3D culture, each well of eight well chamber slides (#154534, Lab-Tek) was precoated with 10 µl of 100% low growth factor Matrigel (#35623, Corning). Five thousand cells suspended in complete media containing 2% Matrigel with or without Mek inhibitor were plated onto each well and then incubated at 37 °C with 5% CO_2_ for 5 days with cell media changed every 2–3 days. During this time, cells developed into cysts and were either used for protein isolation or fixed with 4% paraformaldehyde for morphological analysis or immunofluorescence staining.

Cysts were grouped into three different phenotype categories: single, multiple, and filled lumens. Single lumen cysts were cysts contain one central lumen. Cysts containing multiple lumens displayed many lumens of varying sizes or with one large lumen plus two or more smaller lumens. Cysts with no apparent lumen were classified as cysts containing filled lumens. For statistical analysis, more than 80 individual 3D cysts were counted for each sample in the experiment. Average phenotype percentage ratios of more than three experiments were calculated and *p*-values were obtained by two-tailed *t* test with equal variance.

To compare cyst size between samples, average of vertical and horizontal diameters of 3D cysts were calculated (*n* > 30 for each sample) and *p*-values calculated using two-tailed *t*-test with equal variance.

### Immunofluorescence and immunohistochemical staining

Cells grown on coverslips or in 3D Matrigel were fixed in 4% PFA permeabilized with 0.1% Triton X-100. Primary antibodies were applied to samples on coverslips or in the chamber wells and incubated at 4 °C overnight. After several washes, samples were incubated with secondary antibodies or phalloidin tagged with fluorescence. Images were taken using LSM 500 confocal microscope from Zeiss. Multiple Z sections of the cysts are taken for each 3D spheroid, with stack distance of 0.5 µm taken for each Z stack.

For live-cell imaging of GFP-actin transformed MDCK cells transfected with various tdTomato-TNS1 mutants, images were collected by a Zeiss AxioObserver equipped with a Yokogawa CSU-10 spinning disk confocal system, 488 and 561 nm solid-state lasers, and a Photometrics CoolSNAP HQ camera.

The kidney sections were de-paraffinized in xylene and rehydrated in graded alcohol and water. An antigen retrieval step (10 nM sodium citrate (pH 6.0) at a sub-boiling temperature) was used for each primary antibody. Endogenous peroxidase activity was blocked by 3% hydrogen peroxide, followed by blocking serum and sections were incubated with primary antibodies overnight at 4 °C. Detection of immunostaining was carried out by using the VECTASTAIN® ABC system, according to the manufacturer’s instructions (Vector Laboratories).

### Protein lysate preparation and immunoblot analysis

Cysts grown in Matrigel were recovered using Cell Recovery Solution (Corning) to dissolve the extracellular matrices and then were lysed in ice-cold lysis buffer (20 mM Tris-HCl, pH 7.5, 50 mM NaCl, 1% NP-40, 1% sodium deoxycholate, 2.5 mM sodium pyrophosphate, 1 mM β-glycerophosphate, 1 mM Na3VO4, 1 μg/ml leupeptin, and protease/phosphatase inhibitor cocktail (Roche)) for total protein preparation. Frozen kidneys were first homogenized using a 2-ml Dounce homogenizer (Wheaton) in lysis buffer. Cell/tissue lysates were centrifuged at 14,000 RPM for 15 min at 4 °C and supernatant portions were collected for immunoblotting analysis. Protein concentrations were measured with Protein Assay Dye Reagent Concentrate (Bio-Rad).

For immunoblot analysis, proteins were separated on an SDS-PAGE gel and transferred to nitrocellulose membrane (Bio-Rad). Membranes were incubated in blocking buffer (5% non-fat milk) for 1 h and then with primary antibodies overnight at 4 °C. After several washes, membranes were incubated with secondly antibodies for 1 h at room temperature. The signals were detected by using enhanced chemiluminescence (ECL) reagents (Advansta) and AzureBiosystems C400 chemiluminescent imaging system.

### Mouse studies

TNS1-KO mice were generated as described^23^. Trametinib in corn oil (Sigma-Aldrich) was orally administrated to 3-month-old TNS1-KO and WT mice at 1 mg/kg once daily for 14 days. Four days after the last treatment, mice were euthanized, and kidneys were fixed in 4% paraformaldehyde buffered solution and processed for gross morphological examination, H&E, Sirius Red, and immunohistochemical staining. A portion of the kidney tissues was flash frozen in liquid nitrogen and immediately stored at −80 °C for RNA and protein extraction. At least 4 male mice each group were randomizedly chosen for studies. *N* = 4 is derived from power analysis using the one-sample binomial one-sided exact test in DSTPLAN in order to achieve the design parameters of a significance level of α = 0.05 with 80% power. All mouse studies were performed according to protocols approved by institutional animal care and use committees of the University of California-Davis. Animals were maintained in specific pathogen-free environments.

### Quantification of inflammatory cell infiltration and immunohistochemical signals

Ten random photographs from hematoxylin and eosin (H&E)–stained kidney sections were taken at a magnification of ×200 using an Olympus BX40 microscope with an Olympus DP-72 camera. Inflammatory cell infiltration was evaluated semi-quantitatively by a pathologist blinded to group assignment (arbitrary inflammation score 0–3, where 0 = no change; 1 = mid; 2 = moderate and 3 = severe).

To quantify the immunohistochemical signals from each kidney section, ten random photographs were taken at a magnification of ×100 (Sirius red) or ×200 (pMek, MeK, pERK). These images were analyzed and quantified by ImageJ software version 1.48 as described^27^.

### Statistical analysis

Data were presented either as the mean ± SD or the mean ± SE of at least three independent experiments. All experimental results were included for statistical analysis unless the experiments were not completed successfully, for example mice die unexpectedly, transfection was not successful, or protein gel was not loaded equally. The quantitative in vitro and in vivo data were analyzed using the student’s *t*-test. All analyses were performed using SPSS software (v20.0; SPSS, Inc., Chicago, IL). All statistical tests were two-sided and *P*-values < 0.05 were considered statistically significant.

## Results

### Generation and validation of TNS1-KO MDCK cells

**Fig. 1:**
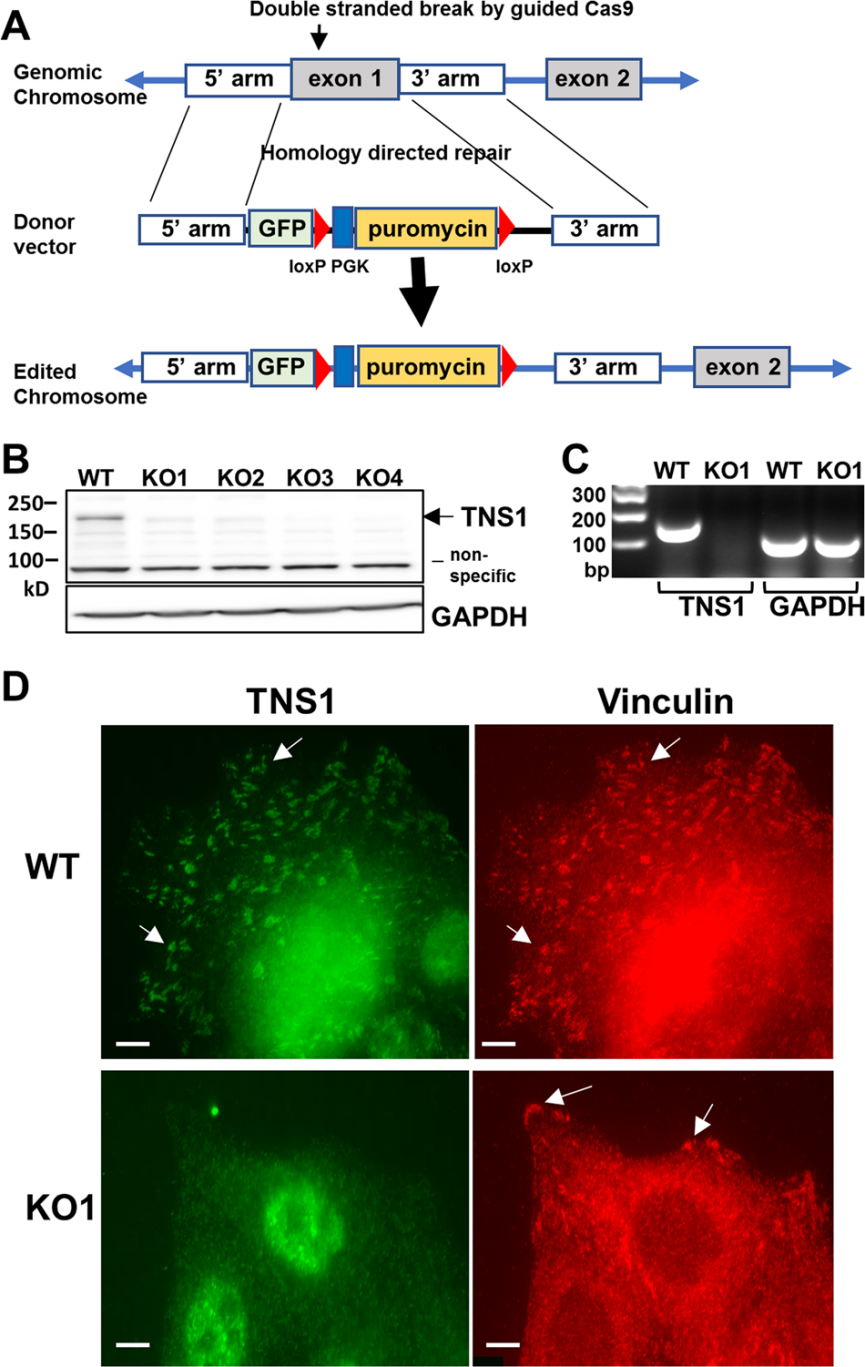
Generation and validation of TNS1-KO MDCK cells. **a** Schematic diagram of the generation of TNS1-KO cells by using two vectors CRISPR/Cas9 and homology-directed repair technology. The guide plasmid containing the TNS1 target sequence and Cas9 coding sequence and the donor vector were co-transfected into cells. The guide plasmid created a double-stranded break at the target site of TNS1. The donor vector contains about 600 bp homologous to the 5′ and 3′ flanking region of exon1 and is used as template for DNA repair by homologous recombination, resulting in replacement of TNS1 exon1, which contains the first ATG site, with the donor cassette that includes promoter-less GFP, PGK promotor and the puromycin selection gene flanked with loxP sites. MDCK WT or KO cells were lysed or fixed for immunoblotting (**b**), RT-PCR (**c**), or immunofluorescence (**d**) assays, respectively. Immunoblotting by TNS1 antibody detected 220kD TNS1 protein in WT but not any of the four KO clones. KO1 clone was used for further analysis. TNS1 mRNA was only presented in WT cells. Immunofluorescence staining showed co-localizations of TNS1 and vinculin at focal adhesion sites (arrows) in WT but not in KO cells. Nuclear stainings shown in WT and KO1 with TNS1 antibody were likely due to the secondary antibody. Scale bar = 10 μm.

To determine mechanistic insights of the polycystic formation in TNS1-KO kidney, we opted to establish an in vitro system and generate MDCK TNS1-KO cells by CRISPR-Cas9 and homology-directed repair (HDR) methodology. The Cas9 vector containing a TNS1 guide sequence targeting the exon encoding the start ATG site of TNS1 and the donor vector containing the promoter-less GFP reporter and puromycin selection cassette flanked by 5′ and 3′ TNS1 homologous regions, were co-transfected into MDCK cells (Fig. 1a). After 7 passages to dilute out the Cas9 vector, puromycin was applied to select stable clones. Individual stable clones were isolated and analyzed for TNS1 expression by immunoblotting (IB), RT-PCR, and immunofluorescence (IF) staining. The lack of 220kD TNS1 protein, mRNA, and the focal adhesion staining pattern (Fig. 1b–d) demonstrate that we have successfully generated MDCK TNS1-KO cells. However, GFP was not detected in any of TNS1-KO stable clones, suggesting that the endogenous TNS1 promoter did not initiate the transcription of GFP gene and/or the level of GFP product was too low to be detected.

### Loss of TNS1 leads to multiple lumen phenotype but maintains the apical-basal polarity in MDCK 3D culture

**Fig. 2:**
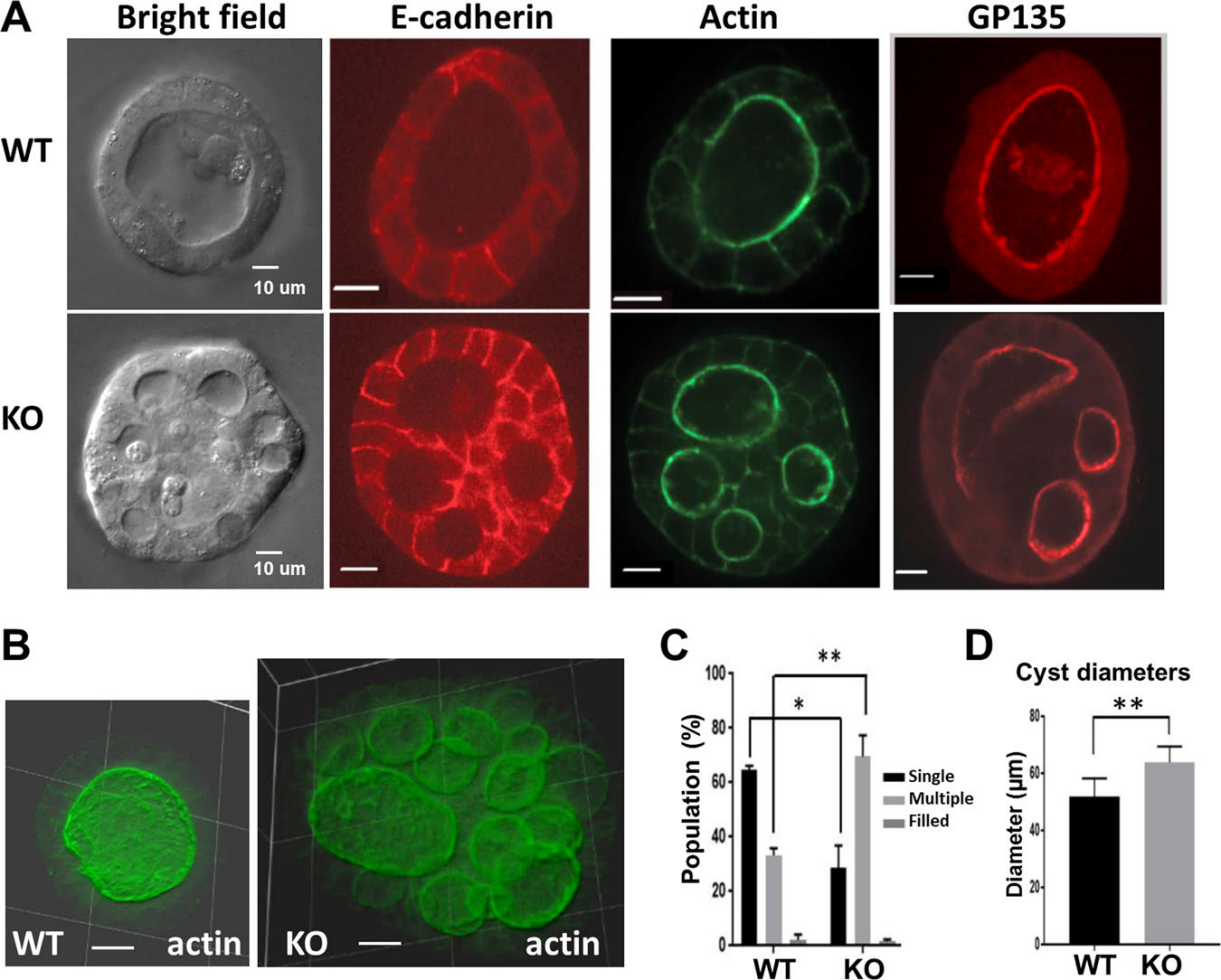
Loss of TNS1 leads to MDCK 3D cysts containing multiple lumens. **a** TNS1-WT or KO MDCK cells were seeded in Matrigel for 5 days and then fixed for general morphology (bright field) or followed by immunofluorescence staining with Alexa Fluor 488 phalloidin for actin, anti-E-cadherin, and anti-GP135 to detect the polarization patterns. **b** Overhead 3D reconstruction of confocal Z-stack images of representative WT and KO 3D cysts stained with phalloidin for F-actin. **c** Quantification of luminal phenotype populations displayed by TNS1-WT and KO MDCK cells (4 independent experiments with *n* > 80 cysts counted for each experiment), as well as **d** average cyst diameter (*n* > 20 cysts measured for each cell line). *P-*values were calculated using two sample *t*-test. *P < 0.01, **P < 0.001. Scale bar = 10 μm.

To investigate TNS1′s role in 3D cyst development, MDCK TNS1-WT or KO cells were cultured in Matrigel for 5 days. Compared to WT cells which predominantly form 3D cysts containing one hollow lumen, cysts from TNS1-KO cells consisted of two or more smaller lumens (Fig. 2a–c), which were more apparent from the 3D reconstruction of confocal Z-stack images (Fig. 2b). Furthermore, quantification comparing the diameters of MDCK TNS1-WT and KO cysts revealed that cysts that lack of TNS1 were significantly larger than WT cysts (KO vs WT = 64 ± 5.9 vs 52 ± 7.2 μm) (Fig. 2d). Immunofluorescence analysis showed that although forming multiple lumens, TNS1-KO cysts still developed apical-basal polarities as judged by GP135 (apical surface marker) and E-cadherin (lateral membrane marker) staining (Fig. 2a).

**Fig. 3:**
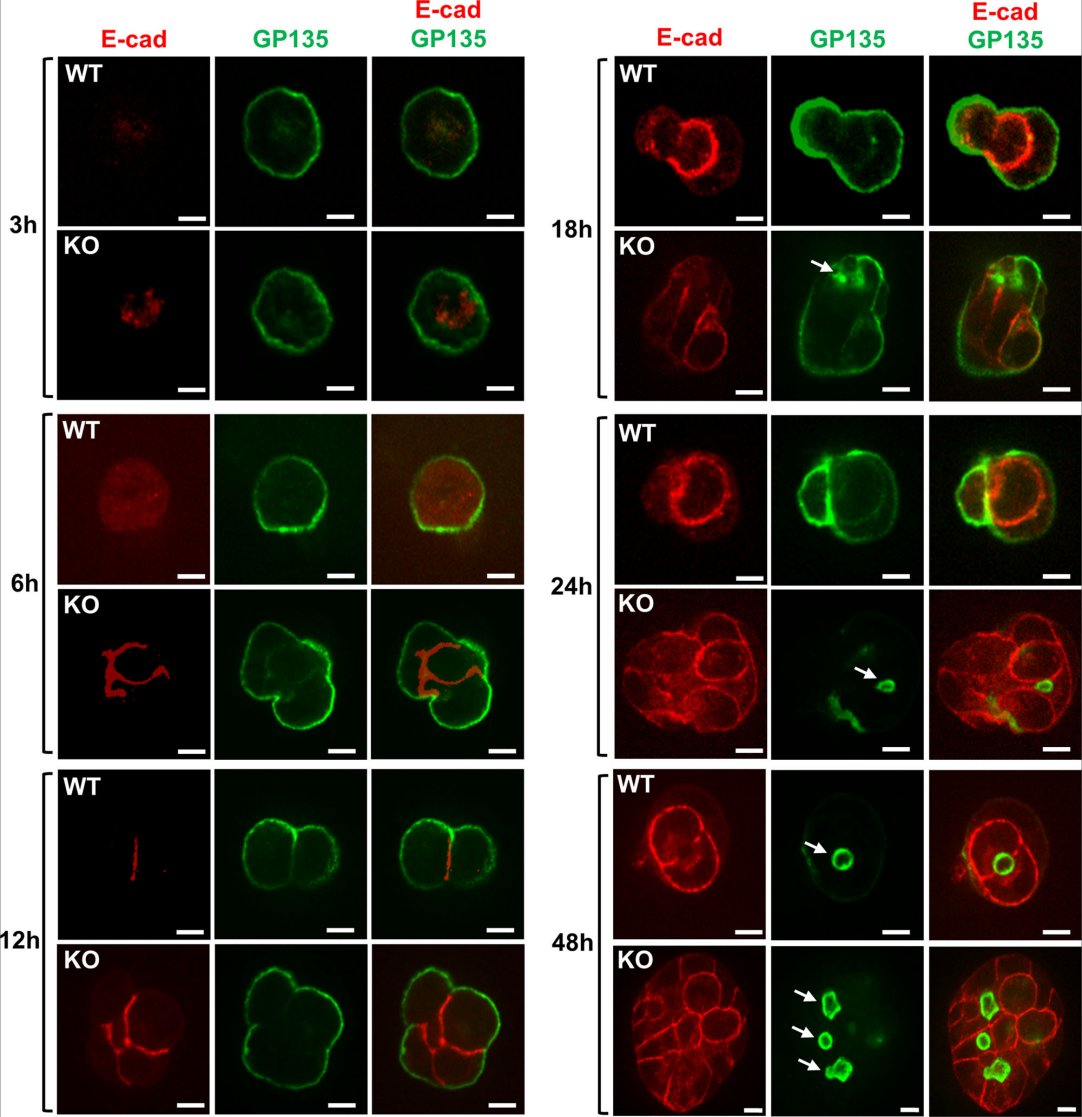
TNS1-KO cells form multiple apical membrane initiation sites early during 3D culture. TNS1-WT or KO MDCK cells cultured in Matrigel for 3, 6, 12, 18, 24 or 48 h were fixed and stained with GP135 and E-cadherin antibodies to detect polarization and cell–cell junction formations during early cyst development. The experiments were repeated three times. Arrows show apical membrane initiation sites. Scale bar = 10 μm.

To examine the onset of multiple lumen formation, MDCK cells grown in Matrigel were harvested at earlier time points for immunofluorescent staining using antibodies against GP135/podocalyzin and E-cadherin. In WT cells, GP135 was initially localized at the basal surface from 3 to 24 h and was then relocated to the newly formed single apical membrane initiation site (AMIS) between 24 and 48 h (Fig. 3). The AMIS matured and later expended into a lumen. E-cadherin staining in the WT cells was absence or diffused at 3–6 h and showed signs of cell–cell junctional pattern around 12 h. In TNS1-KO cells, GP135 started to relocate forming an AMIS at 18 h and was almost completely concentrated at the AMISs at 24 hr, whereas cell–cell junctional E-cadherin could be detected at 6 h (Fig. 3). These results indicate that TNS1-KO cells can still establish the apical-basal polarity and cell–cell junction, and form AMIS, but AMIS in TNS1-KO cells develop much faster than the WT cells and cannot ensure only one AMIS is formed in each cyst. Altogether, our data suggest that TNS1-KO 3D culture mimics the polycystic kidney phenotype observed in TNS1-KO mice.

### Cell–cell junctional localization of TNS1 is required for rescuing multiple lumen phenotypes

**Fig. 4:**
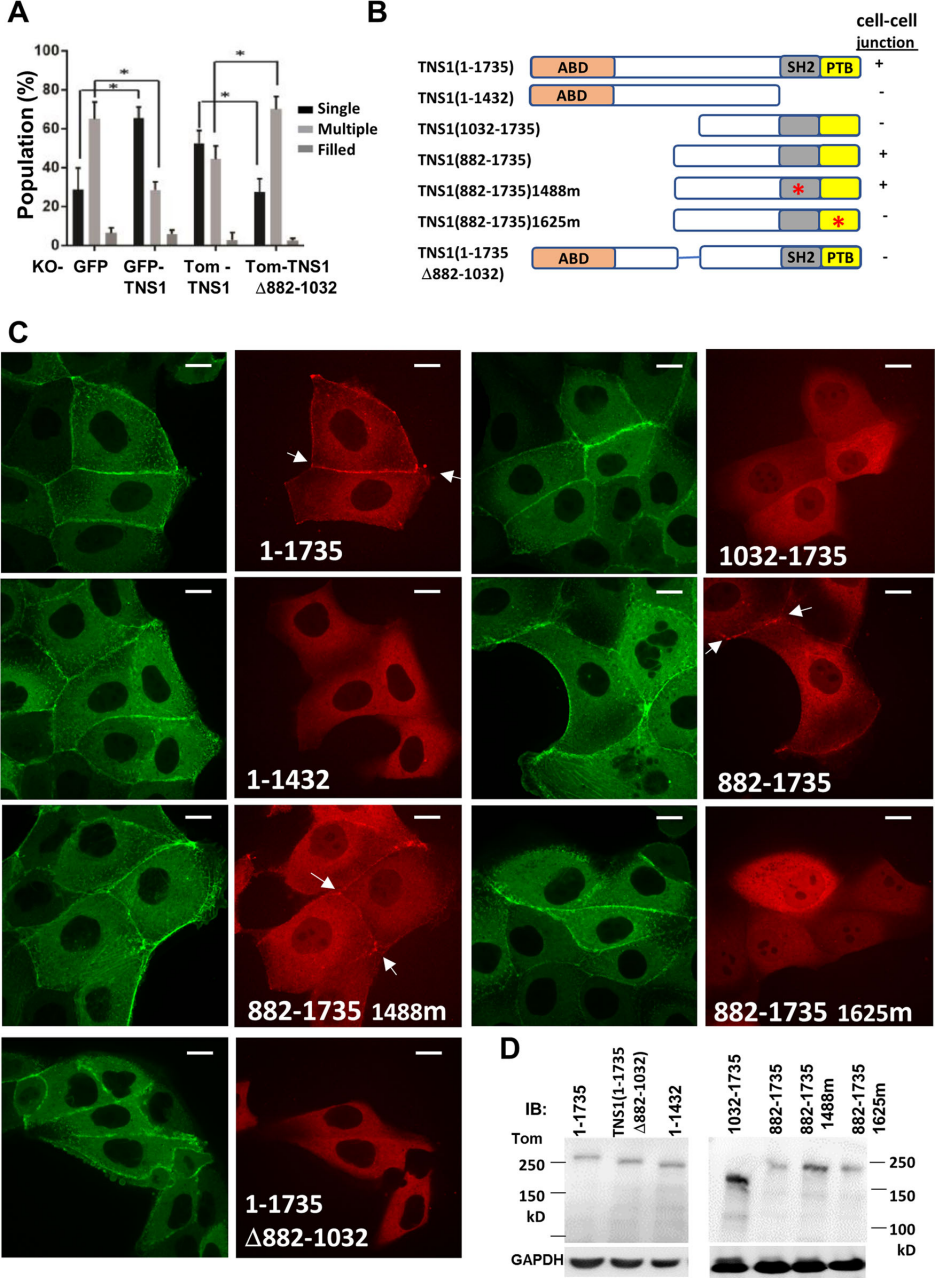
TNS1 localization to cell junctions is critical for single lumen formation. **a** Phenotypic quantification of MDCK KO/GFP, KO/GFP-TNS1, KO/Tom-TNS1(tdTomato-TNS1) and KO/Tom-TNS1Δ882–1032 stable cell lines cultured in 3D Matrigel. *P < 0.01. **b** Schematic diagram of Tom-TNS1 mutants used for identification of primary sequences required for cell–cell localization and summary of localization results. The 1488 m and 1625 m mutants contain point mutations that inactive SH2 and PTB binding activities, respectively. **c** Representative live-cell images of MDCK cells constitutively expressing GFP-actin cells transfected with indicated Tom-TNS1 mutants. Arrows point to cell–cell junctions. **d** Immunoblot analysis confirmed the expression of expected molecular weights of indicated Tom-TNS1 mutants. Scale bar = 10 μm.

To confirm the multiple lumen phenotype is due to lack of TNS1 and to dissect the essential domain required, GFP-TNS1 or tdTomato-TNS1 (Tom-TNS1) were transfected into TNS1-KO MDCK cells to establish TNS1-KO/GFP-TNS1 or TNS1-KO/Tom-TNS1 stable clones, which were analyzed in 3D culture. Re-expression of TNS1 significantly reduced multiple lumens and increased single lumen cysts in 3D Matrigel (Fig. 4a). Since the cell–cell junction is critical in lumenogenesis in MDCK and TNS1 is also known to localize to cell–cell junctions^10^, the significance of TNS1 cell–cell localization to lumen formation was investigated. To this end, we first identified domain/regions required for TNS1 cell–cell junction localization with a series of mutants fused with tdTomato (Fig. 4b–d). We found that (1) the shortest fragment required is aa 882–1735, (2) the functional SH2 domain is not required, (3) the functional PTB domain is needed, and (4) aa 882–1032 region is essential for this subcellular localization (Fig. 4c). We then generated a deletion mutant Tom-TNS1Δ882–1032, which does not localize to cell–cell junctions (Fig. 4c). Re-expression Tom-TNS1Δ882–1032 in KO cells failed to rescue multiple lumen phenotypes of KO cells (Fig. 4a). This demonstrates the critical role of cell–cell junction localization of TNS1 in proper lumen formation in MDCK cells.

### Upregulation of pMek is critical for multiple lumen phenotype in MDCK TNS1-KO 3D culture

**Fig. 5:**
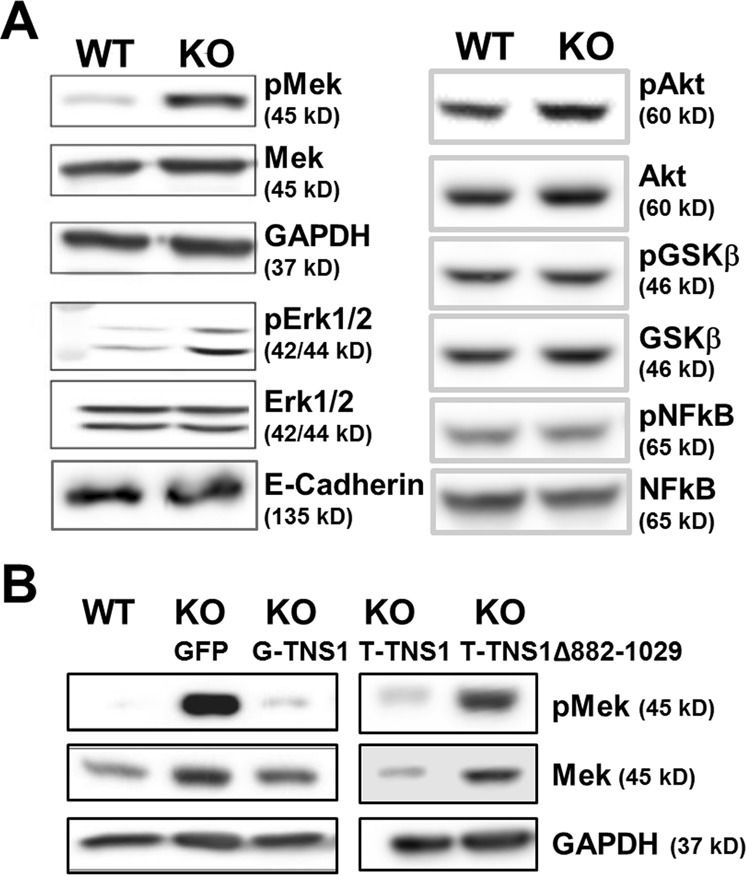
The Mek/Erk pathway is upregulated in TNS1KO 3D cysts. **a** Total cell lysates isolated from 5-day 3D cultures of MDCK TNS1-WT or KO (**a**) or KO/GFP, KO/G(FP)-TNS1, KO/T(omato)-TNS1, KO/T(omato)-TNS1 Δ882-1029 (**b**) were immunoblotted (IB) with indicated antibodies. The studies were repeated at least three times.

**Fig. 6:**
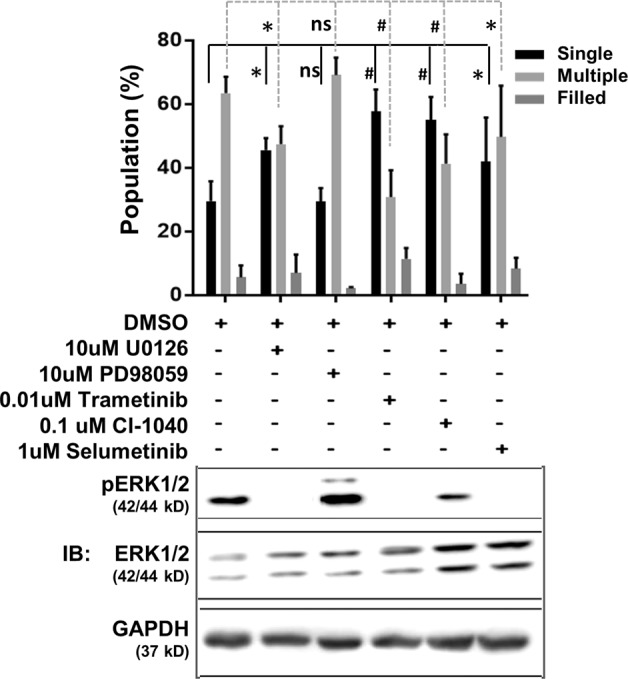
Multiple lumen phenotype in TNS1-KO MDCK cells is reversed by Mek inhibitor treatments. MDCK TNS1-KO cells cultured in 3D matrigel together with indicated reagents for 5 days were analyzed for the populations of single, multiple, and filled lumens (upper panel) or for immunoblotting with pErk1/2, Erk1/2, or GAPDH antibodies (lower panel). The experiments were independently repeated three times. Note that all Mek inhibitors except PD98059 rescued KO lumen phenotypes with trametinib to be the most effective. *P-*values were calculated using two sample *t*-test. *P = 0.02, ^#^*P* < 0.01.

To understand the signaling pathways that may lead to TNS1-KO phenotype, we took a candidate screening approach by immunoblotting. While the levels of pAkt, Akt, pNFkB, NFkB, pGSKβ, GSKβ, and E-cadherin were unaffected, pMek and pErk levels were consistently upregulated in TNS1-KO MDCK 3D culture (Fig. 5a). To demonstrate upregulated pMek was directly related to loss of TNS1, pMek levels were examined in TNS1-KO/GFP-TNS1 and TNS1-KO/tdTomato-TNS1 cells. Re-expression of TNS1 in KO cells reduced pMek levels to that of WT cells (Fig. 5b), indicating that enhanced Mek activity is due to loss of TNS1 and may contribute to multiple lumen phenotype in KO cells. In addition, the Tom-TNS1Δ882–1032 mutant that did not localize to cell–cell junctions (Fig. 4c) and did not rescue multiple lumen phenotypes of KO cells (Fig. 4a) also could not inhibit the upregulated pMek (Fig. 5b), indicating that cell–cell junction localization of TNS1 is required for suppressing Mek activity. To further evaluate the role of Mek activity, various Mek inhibitors were used to test whether they could reverse multiple lumen phenotypes in TNS1-KO MDCK. While U0126 (10 μM), trametinib (0.01 μM), selumetinib (1 μM), CI-1040 (0.1 μM) rescued the multiple lumen phenotypes, PD98059 (10 μM) treatment showed no effect (Fig. 6). Interestingly, immunoblotting analysis confirmed that all inhibitors except PD98059 were able to reduce levels of pErk, the immediate target of Mek (Fig. 6). This may explain why PD98059 treatment has no rescue effect.

### pMek is upregulated in TNS1-KO kidneys and Mek inhibitor treatment reduces disease burden in TNS1-KO mice

**Fig. 7:**
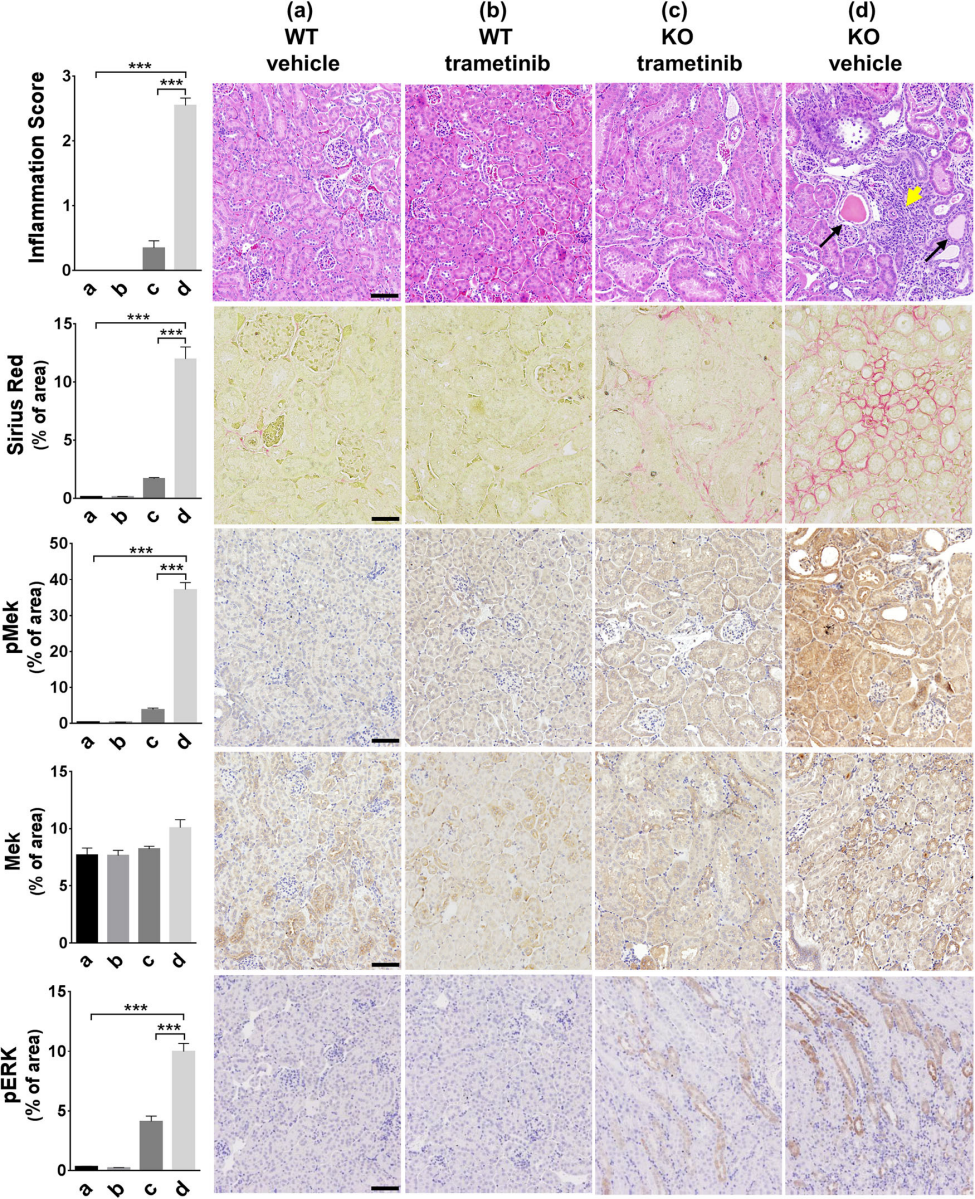
Mek inhibitor treatment reduced the levels of upregulated pMek, pErk, interstitial infiltrates, tubular dilations, and fibrosis in TNS1-KO kidneys. TNS1-WT or KO male C57 mice (*N* = 4, each group) at 3-month-old were treated with trametinib (1 mg/kg) or vehicle for 14 days. Kidneys were harvested 4 days post-treatment and processed for H&E, IHC staining using pMek1/2(pS221), Mek1/2, or Erk(pT202pY204) antibodies, and Sirius Red staining for collagen. Interstitial infiltration and IHC signals were quantified for statistical analysis shown on the left panel. Note that levels of pMek and pErk, and signs of interstitial infiltrates, fibrosis and dilated tubules were significantly reduced in TNS1-KO kidneys treated with trametinib. Arrows show dilated tubules. Arrowhead indicates interstitial infiltrates. Scale bars = 50 μm in Sirius Red, or 100 μm in others. *P*-values were calculated using two sample *t*-test. ***P < 0.0001.

Our studies using MDCK TNS1-KO cells have pointed to a critical role of upregulated Mek activity as judged by pS221-Mek levels (Fig. 5). To validate this finding in the kidneys, kidney sections from 3-month old TNS1-WT or KO mice were stained with pMek (S221), Mek, and pErk(T202Y204) antibodies. In agreement with in vitro studies, pMek and pErk levels were significantly increased in the KO over the WT kidneys (Fig. 7, WT vehicle vs KO vehicle). To further test whether Mek inhibitor treatment may reduce the disease burden, we treated 3-month old WT and KO mice with trametinib (1 mg/kg orally once a day for 14 days), the most effective inhibitor shown in our MDCK studies (Fig. 6). Kidneys were collected 4 days post treatment and prepared for H&E and IHC staining assays. With trametinib treatment, pMek and pErk levels were reduced, and signs of interstitial infiltrates, fibrosis and dilated tubules were significantly improved in TNS1-KO kidneys (Fig. 7). These results not only validate our in vitro findings but also provide a potential therapeutic strategy using Mek inhibitors for cystic kidney diseases.

## Discussion

In this report, we have established a 3D MDCK TNS1-KO cell system, which develops into cysts with multiple lumens that mimic cystic kidney defects observed in TNS1 knockout mice. This in vitro system allows us to further identify the critical roles of a TNS1 fragment (aa 882–1032) that is required for TNS1′s cell–cell junction localization, for suppression of the Mek/Erk activities, and for rescuing the multiple lumen phenotype.

Cell–cell junctions play important roles in kidney morphogenesis and functions. In MDCK cells, down-regulation of adherens junction molecule such as E-cadherin, or tight junction molecules including ZO1 or ZO2 lead to compromised 3D cysts with multiple lumens^28–30^ similar to those of TNS1-KO cysts. These findings clearly demonstrate the critical role of cell–cell junction in lumenogenesis. It is known that knockdown of E-cadherin activates Mek/Erk signaling and promotes epithelial-mesenchymal transition (EMT)^31^. Cleavage of E-cadherin also enhances Mek pathway^32^. Although the cell junction localization and protein level of E-cadherin appear to be normal in TNS1-KO cysts (Figs. 2a and 5a), it is possible that cell junction localization of TNS1 may suppress Mek/Erk signaling through stabilizing the integrity of cell–cell junction.

Although our current studies have demonstrated the essential role of cell–cell junction localization in directing the single luminal development, the potential contribution of TNS1′s focal adhesion localization in this process has not been ruled out. Because TNS1 contains at least two independent focal adhesion targeting sites, one overlapping with an actin-binding region and the other within the SH2 and PTB domains^33^, it is more challenging to dissect their direct involvement in the multiple lumen phenotype. Nonetheless, we are continuing to study the possibility.

Upregulation of Mek/Erk activities appear to be critical for developing multiple lumen phenotypes in TNS1-KO cells, since Mek inhibitor treatments could rescue the phenotypes. This finding not only prompted us to examine pMek/pErk levels in TNS1-KO kidneys but also to investigate the potential therapeutic effects of using Mek inhibitors in TNS1-KO mice. Our current studies indicate that trametinib treatment is able to reduce pathogenesis of TNS1-KO kidneys in the early stage. Long term beneficial effects and optimal treatment regimen warrant further investigation. Interestingly, upregulation of Mek/Erk activity is also detected in the cystic kidneys of polycystin-1 knockout mice and polycystin-2 transgenic mice^34–38^. Additionally, primary cell cultures from human ADPKD kidneys also display upregulated Erk activities^34^. Previously, Mek inhibitors were used to treat PKD mouse models. While PD184352 treatment slows polycystic kidney progression in *pcy* mice^38^, U0126 treatment shows no effect on progression of cyst formation in the PKD1 model^35^. Whether the discrepancy is due to using different kinds of inhibitors and/or mouse models require further investigation. Nonetheless, with our findings, targeting the Mek/Erk pathway remains a promising therapeutic approach for cystic kidney diseases.

Currently, there is no known human cystic kidney patient that is caused by TNS1 mutation. Nonetheless, because TNS1-KO mice develop cystic phenotypes progressively, are fertile, and live almost half of the normal mouse lifespan, it is very likely that a group of human cystic kidney patients display similar symptoms caused by mutations or aberrant expression of TNS1. These patients are also likely to suffer from mitral valve prolapse (MVP) and/or chronic obstructive pulmonary disease (COPD), since TNS1 is a high-risk gene for both health issues identified through genome-wide association studies (GWAS)^24,39,40^. We are in the process of collecting and sequencing patient samples to test this possibility.
